# Oxygen, carbon dioxide, and nitric oxide: role in the pathophysiology of obstructive sleep apnea

**DOI:** 10.4103/mgr.MEDGASRES-D-25-00224

**Published:** 2026-09-12

**Authors:** Antonio Fabozzi, Pasquale Tondo, Caterina Antonaglia, Alessia Steffanina, Donato Lacedonia, Marco Confalonieri, Matteo Bonini, Paolo Palange

**Affiliations:** 1 Department of Public Health and Infectious Diseases, Pulmonology Unit, Policlinico Umberto I, “Sapienza” University of Rome, Rome, Italy; 2 Department of Medical and Surgical Sciences, University of Foggia, Foggia, Italy; 3 Respiratory and Intensive Care Unit, University-Hospital Policlinico of Foggia, Foggia, Italy; 4 Pulmonology Unit, Department of Medical Surgical and Health Sciences, Hospital of Cattinara, University of Trieste, Trieste, Italy

**Keywords:** carbon dioxide, cardiovascular disease, cognitive dysfunction, hypercapnia, hypoxia, inflammation mediator, metabolic syndrome, nitric oxide, obstructive sleep apnea, oxygen

## Abstract

Obstructive sleep apnea is a prevalent sleep-related breathing disorder with recurrent episodes of upper airway collapse during sleep, leading to cyclic blood gas fluctuations. Oxygen, carbon dioxide, and nitric oxide play a crucial role in the pathophysiology. This narrative review aims to explore the role of gases in obstructive sleep apnea pathophysiology, focusing on the molecular pathways and effects of chronic intermittent hypoxia, intermittent hypercapnia, and nitric oxide dysregulation. Chronic intermittent hypoxia stimulates molecular pathways such as hypoxia-inducible factor-1α and inflammatory cascades such as nuclear factor kappa B, leading to oxidative stress, endothelial dysfunction, and sympathetic overactivation. Intermittent hypercapnia impacts on chemoreflex sensitivity, ventilatory instability, and autonomic function, with emerging evidence of both pathogenic and potentially beneficial effects. Nitric oxide, which modulates airway tone, vascular reactivity, and immune control, presents a reduced bioavailability in patients with obstructive sleep apnea. These three gas mediators contribute to the complex pathophysiology of obstructive sleep apnea and underlie the long-term cardiovascular, metabolic, and neurocognitive risks of obstructive sleep apnea. Appropriately prioritizing the relationship between these gases and the pathophysiology of obstructive sleep apnea contributes to a new understanding of the disease and new treatment approaches in the future.

## Introduction

Obstructive sleep apnea (OSA) is a chronic respiratory disease whose incidence is steadily increasing. It is characterized by recurrent episodes of partial or complete obstruction of the upper airways during sleep, resulting in intermittent hypoxia, fragmented sleep and marked fluctuating intrathoracic pressure.^1^,^2^,^3^ Recent epidemiological estimates indicate that approximately one billion adults worldwide are affected by OSA, of whom more than 400 million suffer from moderate-to-severe disease, the forms most strongly associated with cardiovascular events, mortality and healthcare costs.^4^ Despite its high prevalence and systemic consequences, OSA remains largely underdiagnosed and undertreated, reinforcing its relevance as a major global public health challenge.^1^,^4^ While nocturnal fluctuations in blood oxygen (O_2_) (intermittent hypoxia) are well known for their contribution to inflammation, sympathetic hyperactivity and oxidative stress,^5^ the pathophysiological role of nocturnal fluctuations in blood carbon dioxide (CO_2_) (intermittent hypercapnia) is gaining recognition for its role in ventilatory instability and metabolic consequences.^6^,^7^,^8^,^9^,^10^,^11^,^12^,^13^,^14^,^15^,^16^ Furthermore, nitric oxide (NO), a crucial gas for vascular tone, inflammation, and airway tone, is increasingly emerging as a pivotal mediator in OSA-related systemic dysfunctions.^17^,^18^,^19^,^20^,^21^,^22^,^23^,^24^

This narrative review explores the roles of O_2_, CO_2_ and NO in the pathophysiology of OSA, with a particular focus on their cyclic fluctuations during sleep. We will explore molecular pathways and clinical implications of intermittent hypoxia, intermittent hypercapnia, and the pathophysiological role of NO. In this way, we plan to provide a comprehensive review for identifying the consequences mediated by gases in OSA and suggesting new targets for therapeutic interventions.

## Search Strategy

A comprehensive literature search was performed at Medline and Scopus databases without timeline restrictions. Search terms included obstructive sleep apnea, intermittent hypoxia, intermittent hypercapnia, nitric oxide, oxidative stress, inflammation, endothelial dysfunction, and sympathetic activation. We included experimental studies (animal and human models), clinical observational and interventional studies, and high-quality narrative or systematic reviews.

## Role of Oxygen in Obstructive Sleep Apnea

Chronic intermittent hypoxia (CIH) plays a key role in the pathophysiological consequences of OSA, triggering a series of cellular, molecular, and systemic abnormalities. Unlike prolonged hypoxia, CIH causes recurrent cycles of desaturation and reoxygenation that trigger inflammatory, oxidative, and autonomic mechanisms, making it particularly dangerous.^5^

CIH is known to induce sympathetic nervous system (SNS) overactivity, oxidative stress, systemic inflammation, metabolic dysfunction, endothelial dysfunction, and epigenetic reprogramming, thereby leading to a variety of clinical conditions, such as hypertension, cardiac arrhythmias, atherosclerosis (AS), pulmonary hypertension, cognitive impairment, and tumor progression.^25^,^26^,^27^,^28^,^29^

### Molecular mechanisms of chronic intermittent hypoxia

The family of transcription factors known as hypoxia-inducible factor (HIF) plays a key role in the molecular pathways induced by CIH (**Figure 1**). Under normoxic conditions, HIF-1α is hydroxylated by prolyl hydroxylase domain proteins, which determine its degradation through the von Hippel-Lindau ubiquitin-proteasome pathway.^30^ During hypoxia, prolyl hydroxylase domain protein’s function is suppressed, leading to greater stabilization of HIF-1α. Stabilized HIF-1α translocates to the nucleus, binds to hypoxia-responsive elements and induces transcriptional upregulation of hypoxia-sensitive genes implicated in angiogenesis (such as vascular endothelial growth factor), metabolism (such as glucose transporter 1), inflammation (such as interteukin (IL)-6), and erythropoiesis (such as erythropoietin).^31^ Experimental animal models have shown that exposure to CIH significantly increases HIF-1α and reactive oxygen species (ROS) production of up to 70%.^32^

**Figure 1:**
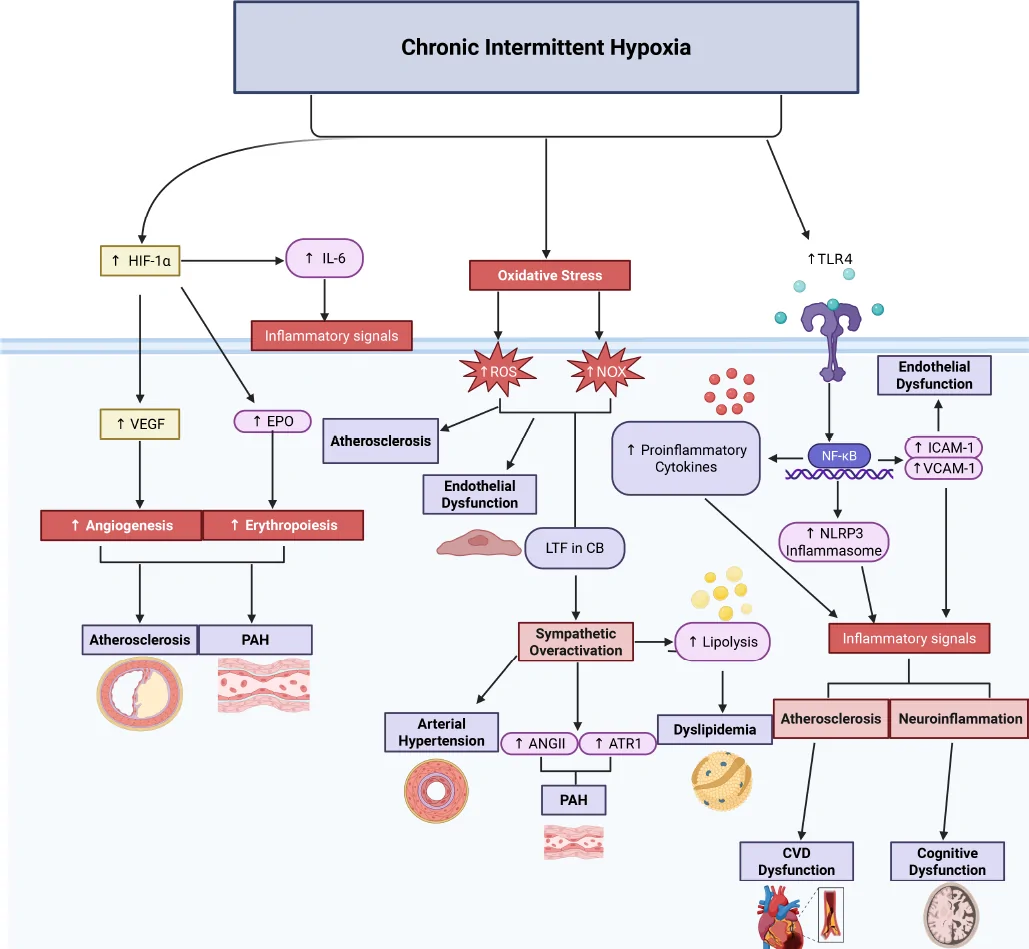
Chronic intermittent hypoxia: from molecular pathway to clinical implication. Chronic intermittent hypoxia activates HIF-1α, inducing VEGF-mediated angiogenesis, EPO mediated erythropoiesis and IL-6-dependent inflammation. Enhanced angiogenesis and erythropoiesis lead to atherosclerosis and PAH. Chronic intermittent hypoxia also increases ROS and NOX, which contribute to the pathogenesis of atherosclerosis, endothelial dysfunction and sympathetic overactivation (through LTF in CB). Sympathetic overactivation induces arterial hypertension, dyslipidemia (which induces lipolysis) and PAH (through ANGII and ATR1). Chronic intermittent hypoxia also stimulates TLR4-NF-κB pathway, which activates NLRP3 inflammasome and proinflammatory cytokines, inducing CVD through atherosclerosis and cognitive dysfunction through neuroinflammation. NF-κB also activates ICAM-1 and VCAM-1, contributing to endothelial dysfunction. Created with BioRender.com. ANGII: Angiotensin II; ATR1: angiotensin II type-1 receptor; CB: carotid body; CVD: cardiovascular dysfunction; EPO: erythropoietin; HIF-1α: hypoxia-inducible factor-1α; ICAM-1: intercellular adhesion molecule 1; IL: interleukin; LTF: long-term facilitation; NF-κB: nuclear factor kappa B; NLRP3: NOD-like receptor protein 3; NOX: nicotinamide adenine dinucleotide phosphate oxidase; PAH: pulmonary arterial hypertension; ROS: reactive oxygen species; TLR4: Toll-like receptor 4; VCAM-1: vascular cell adhesion molecule 1; VEGF: vascular endothelial growth factor.

Recurring episodes of hypoxia and reoxygenation during CIH, mimicking the ischemia-reperfusion cycle, are known to increase ROS production.^33^ These ROS not only act as damaging oxidants but also serve as redox signaling molecules that influence gene transcription and cellular adaptation.^27^ Another finding which supports the key role of ROS in CIH-induced pathophysiology comes from the administration in animal models of a powerful superoxide scavenger, manganese (III) tetrakis (1-methyl-4-pyridyl) porphyrin pentachloride, which reduces the effects of CIH on the carotid body (CB) sensitivity.^34^ CIH, through ROS-mediated pathway, induces increased structural and functional plasticity in the CB, causing a higher sensitivity to hypoxia and enhanced release of neurotransmitters from glomus type 1 cells.^35^ Notably, Ca^2+^/calmodulin-dependent signaling has emerged as a key ROS-induced mechanism in CIH. In fact, the use of calcium chelators inhibited the activation of HIF-1α by CIH.^36^ Furthermore, clinical studies conducted on OSA patients have identified circadian fluctuations in serum and urinary biomarkers linked to CIH, highlighting their potential role as non-invasive markers of disease status and therapeutic response.^37^ For example, levels of vascular endothelial growth factor, endothelin-1, and oxidative stress markers, which are elevated in OSA patients due to exposure to CIH, decrease with the initiation of continuous positive airway pressure (CPAP) therapy.^38^,^39^,^40^

### Chronic intermittent hypoxia and inflammation

CIH induces systemic and local inflammation through complex pro-inflammatory molecular pathways. Park et al.^41^ demonstrated that CIH significantly increases Th17 cell populations and concomitantly reduces regulatory T cells in a murine model. Furthermore, the elevated levels of IL-17A and IL-4 further highlight the importance of the Th17-mediated inflammatory pathway in the pathophysiology of CIH-induced inflammatory injury.

Toll-like receptor (TLR) 4 signaling also plays an important role in the pathophysiology of CIH-induced inflammatory damage. CIH upregulates TLR4 expression, which in turn activates downstream effectors such as nuclear factor kappa B (NF-κB).^42^ NF-κB provokes the overexpression of various proinflammatory cytokines, including tumor necrosis factor alpha (TNF-α), IL-6, IL-8, IL-1β, intercellular adhesion molecule-1, and vascular cell adhesion molecule-1.^42^ These findings are also confirmed by animal studies in which atorvastatin decreased NF-κB levels by reducing TLR4 expression, consequently weakening the inflammatory pathways induced by CIH.^43^

Mitogen-activated protein kinase (MAPK) is a downstream effector pathway of TLR4 and also plays an important role in CIH-induced inflammatory injury. In fact, CIH activates two downstream components of MAPK, p38 and extracellular signal-regulated kinases, through phosphorylation.^42^ These are involved in CIH-induced inflammation in the liver, brain, and myocardium.^44^,^45^,^46^ Further evidence confirming the role of MAPK in the pathophysiology of CIH-induced inflammatory injury comes from the effectiveness of reducing CIH-related inflammation by suppressing the MAPK pathway.^45^,^46^,^47^,^48^

MicroRNAs, which regulate gene expression, are also involved in the pathophysiology of CIH-related inflammatory damage. In fact, experimental studies have demonstrated that upregulated levels of miR-210 are associated with endothelial dysfunction secondary to CIH.^49^ miR-21-5p under-expression in CIH induces overactivity of its target pro-inflammatory genes such as TNF-α, HIF-2α, IL-6, IL-8, TLR4.^50^ Furthermore, miR-155 and miR-210 also promote the expression of the nucleotide-binding domain-like receptor protein 3 (NLRP3) inflammasome.^51^,^52^

CIH increases NLRP3 inflammasome activity, which in turn is associated with inflammatory injury, oxidative stress, and apoptosis in the cardiovascular system through the release of pro-inflammatory cytokines such as IL-1β and IL-18.^53^,^54^

In conclusion, these findings suggest that CIH is a driver of chronic inflammation related to OSA, through immune dysregulation, inflammatory signaling cascades, and epigenetic modulation.

### Chronic intermittent hypoxia and the sympathetic nervous system

It is well known in the literature that CIH can provoke hyperactivity of the SNS, as demonstrated by studies in which CIH is associated with high muscle sympathetic nerve activity (MSNA) and high levels of plasma catecholamines.^55^,^56^ There are multiple mechanisms for this hyperactivation, which are not fully understood.

A key role is played by the CB, whose chemosensitivity to hypoxia increases during CIH.^57^ Experimental studies in mice have shown that CIH leads to a persistent increase in basal CB activity for up to 60 minutes after CIH exposure.^58^ This phenomenon is called “long-term facilitation” (LTF) and indicates a chronic increase in basal sympathetic activity (especially of the CB) in patients with CIH, even in the presence of normoxia.^59^ CIH-induced LTF is reversible. Ten days of normoxia result in the resolution of LTF and of CB increased chemosensitivity to hypoxia.^60^ Elevated expression of endothelin-1 and endothelin-A receptors secondary to CIH contributes to the CIH-induced increases in CB chemosensitivity to hypoxia in rat models.^61^,^62^ Indeed, the use of Bosentan, an endothelin-A receptor antagonist, suppresses the increased chemosensitivity to hypoxia of CB in rat models.^63^ From a molecular perspective, CIH is associated with high levels of ROS in CB, and reduction of ROS levels in CB using ROS scavengers led to suppression of CIH-induced increased chemosensitivity to hypoxia and LTF of CB.58 Nicotinamide adenine dinucleotide phosphate (NADPH) oxidase (NOX) enzyme family plays a crucial role in boosting ROS generation by CB glomus cells after CIH exposure.^64^ In fact, NOX inhibition suppresses CIH-induced LTF of CB.^64^

SNS also plays a key role in CIH-induced arterial hypertension in OSA patients. Tamisier et al.^65^ demonstrated that healthy humans exposed to 14 nights of intermittent hypoxia developed daytime hypertension directly proportional to MSNA hyperactivity, which was in turn induced by CIH. Animal studies showed that pharmacological suppression of CIH-induced MSNA hyperactivity resolved arterial hypertension.^66^,^67^ Furthermore, CPAP therapy reduced blood pressure in OSA patients by blocking CIH-induced MSNA hyperactivity.^68^ The CIH-induced sympathetic excitation, which causes high blood pressure, is mediated by CB. Del Rio et al.^69^ showed that surgical removal of the CB in mice before exposure to CIH prevented them from developing high blood pressure secondary to CIH. In addition, Gilmartin et al demonstrated that 28 nights of CIH exposure in healthy humans was also associated with impaired peripheral vascular reactivity (significantly higher forearm vascular resistance in CIH-exposed subjects).^55^ They proposed that this impaired peripheral vascular reactivity contributed to the increase in blood pressure found in CIH-exposed patients.

Together, CIH induces a chronic sympathetic activation through carotid body-dependent mechanisms, leading to arterial hypertension and vascular dysfunction.

Chronic intermittent hypoxia and cardiovascular dysfunction

It is well known in the literature that CIH accelerates the development of AS in OSA patients, increasing the risk of major cardiovascular events and cerebrovascular events.^5^ First, it should be noted that OSA (and therefore CIH) and AS share common risk factors, including obesity, dyslipidemia, and arterial hypertension.^5^ An experimental study on mice has shown that exposure to CIH for 4 or 12 weeks accelerates the process of AS plaque formation in the aorta.^70^ Elevated levels of erythropoietin induced by CIH also contribute to AS pathogenesis.^71^ A key mechanism of CIH-induced AS is mediated by inflammatory pathways that are overexpressed in CIH. Song et al.^72^ demonstrated that CIH-induced overexpression of NF-κB is involved in the pathogenesis of aortic AS plaque formation. Indeed, suppressing the NF-κB activation pathway slowed down AS. CIH-induced ROS production is another pathogenic mechanism of CIH-induced AS. In fact, suppression of metallothionein, an endogenous ROS scavenger, increased CIH-mediated aortic fibrosis and inflammation.^73^ CIH-induced AS is also stimulated by elevated levels of angiopoietin-like 4 in mice exposed to 4 weeks of CIH, with a higher AS plaque area and higher plasma total cholesterol levels.^74^ CIH also induces endothelial cell dysfunction and endothelial vascular damage, a precursor to AS formation.^75^ In fact, OSA patients often exhibit higher serum levels of intercellular adhesion molecule-1 and vascular cell adhesion molecule-1, which are typically involved in endothelial vascular damage.^76^ CIH-induced endothelial cell dysfunction is a NOX-dependent mechanism.^77^

CIH-induced cardiovascular dysfunction has been demonstrated in numerous experimental animal studies. For example, Morand et al.^78^ demonstrated that mice exposed to 14 days of CIH exhibited a higher incidence of myocardial ischemia-related ventricular arrhythmias, particularly ventricular fibrillation. Wei et al.^79^ demonstrated that mice exposed to CIH for 8 weeks showed reduced mitochondrial respiration and adenosine triphosphate (ATP) production and increased mitochondrial fragmentation in hypertrophic left ventricular cardiomyocytes. The mice showed reduced diastolic function and accumulation of collagen I deposited in the heart. CIH-induced sympathetic overactivity contributes to the pathophysiology of CIH-induced cardiovascular dysfunction. Bizanti et al.^80^ demonstrated that CIH increased sympathetic fiber innervation at the sinoatrial node, atrioventricular node, and major blood vessels connected to the atria. These findings highlight that CIH causes cardiac autonomic imbalance, contributing to CIH-induced cardiovascular dysfunction. In addition, CIH reduced coronary artery flow velocity reserve and caused left anterior descending coronary artery dysfunction.^81^

Overall, these findings indicate that CIH accelerates AS and myocardial injury in OSA, primarily through inflammation, oxidative stress, endothelial dysfunction, and sympathetic hyperactivity.

### Chronic intermittent hypoxia and pulmonary hypertension

CIH contributes to the pathophysiological mechanisms of OSA-related pulmonary arterial hypertension (PAH). Fagan et al.^82^ demonstrated elevated pulmonary artery pressure, right ventricular hypertrophy, and muscle fiber hypertrophy in the distal pulmonary arteries of mice exposed to CIH. The likely mechanism of the relationship between CIH and PAH is NOX-mediated, as mice exposed to CIH but with NOX suppressed did not develop PAH.^83^ Song et al.^84^ identified the role of ROS in the pathogenesis of CIH-induced PAH in mice deprived of manganese superoxide dismutase, an endogenous antioxidant. In these mice, there was an altered remodeling of the pulmonary vasculature proportional to the increased oxidative stress, contributing to the elevation of pulmonary artery pressure.

A recent experimental study in mice demonstrated that CIH upregulates a hypoxia-induced transcription factor, differentially expressed in chondrocytes (DEC1), which suppresses the expression of peroxisome proliferative activated receptor-γ (PPARγ) (a protective factor for PAH), stimulating oxidative stress and inflammation in pulmonary arterial smooth muscle cells and leading to increased risk of PAH.^85^ Recent evidence suggests the roles of CB sympathetic overactivation and the renin-angiotensin system in CIH-induced PAH pathogenesis. A recently proposed model suggests that CIH, by increasing circulating levels of angiotensin II and the expression of angiotensin II receptor type 1 in the CB, increases pulmonary vascular resistance and therefore increases the risk of PAH.^86^

In conclusion, these mechanisms support the role of CIH in the pathophysiology of PAH OSA-related, through the combination of oxidative stress, transcriptional dysregulation, and neurohumoral activation.

Chronic intermittent hypoxia and cognitive dysfunction

CIH is known to induce neurological impairment in OSA subjects, particularly cognitive dysfunction and hippocampal damage.^87^,^88^ CIH causes neural injury through multiple mechanisms, including neuroinflammation, ferroptosis induction, neuroprotective factor suppression, and increased blood-brain barrier (BBB) permeability.^89^,^90^,^91^,^92^,^93^,^94^,^95^,^96^,^97^,^98^,^99^,^100^,^101^,^102^,^103^

From a neuroinflammatory perspective, CIH causes neuronal injury through the activation of inflammatory pathways such as ROS, NOX, NLRP3 inflammasome, and NO synthase (NOS), leading to oxidative stress, neuroinflammation, and endothelial vascular dysfunction.^89^,^90^ Dong et al.^91^ demonstrated that CIH-exposed mice exhibited cognitive deficits due to exaggerated microglial activation and reduced hippocampal expression of PPARγ, alongside increased hippocampal levels of TNF-α and IL-1β. Furthermore, Smith et al.^92^ identified cortical overexpression of TLR4 as one of the primers of CIH-induced neuroinflammation. Even the TLR2-myeloid differentiation primary response 88 inflammatory pathway plays a key role in CIH-induced neuroinflammation, as shown by Li et al.^93^ where mice deprived of TLR2 alleviated cognitive dysfunction and microglial overactivation in the hippocampus. In addition to neuroinflammation, CIH promotes oxidative stress through an iron-dependent oxidative cell death, contributing to neuronal injury by amplifying ROS production.^94^

Furthermore, CIH induces chronic low-grade neuroinflammation in the dorsal hippocampal regions of CIH-exposed mice. This mechanism supports a CIH-induced long-term alteration in microglia, which remains chronically active.^95^ CIH-induced cognitive dysfunction appears to result from impaired hippocampal lipid metabolism in CIH-exposed mice. Li et al.^96^ demonstrated in vitro that CIH increases hippocampal levels of numerous lipids, such as glycerophospholipids, prenol lipids and sphingolipids. Furthermore, CIH causes an accumulation of lipid droplets in the hippocampus. This impaired hippocampal lipid metabolism contributes to neuronal damage and is likely to CIH-induced cognitive dysfunction.

In an animal experimental study, Xie et al.^97^ found significantly lower levels of brain-derived neurotrophic factor in mice exposed to CIH, and this may play a key role in the pathogenesis of hippocampal neuronal damage from CIH. Neuron-specific enolase, a marker of neuronal death and injury, was found to be higher in medullary cells of CIH-exposed mice.^98^ Many studies have shown that elevated levels of proinflammatory cytokines, by inducing neuroinflammation, cause endothelial cell dysfunction and thus disrupt BBB integrity and permeability.^99^ In addition, proinflammatory cytokines can directly access the brain, where the BBB is discontinuous (the circumventricular regions).^100^ Furthermore, proinflammatory cytokines can also attract macrophages, inducing them to migrate to the hippocampus, where they can contribute to the destruction of BBB integrity.^101^ CIH disrupts BBB vascular permeability by impairing the protein transporters/channels present on the BBB.^102^ In fact, Baronio et al.^103^ demonstrated that CIH reduced aquaporin 1 levels in the hippocampus, associating it with cognitive dysfunction.

Overall, it demonstrates that CIH contributes to cognitive dysfunction in OSA, through neuroinflammation, suppression of neuroprotective pathways, and increased BBB permeability.

### Chronic intermittent hypoxia and metabolic dysfunction

From a perspective of glucose metabolism, CIH leads to increased insulin resistance and glucose intolerance in obese mouse models.^104^ CIH stimulates lipolysis and degradation of triglycerides stored in adipocytes by hyperactivating the SNS, resulting in increased release of circulating free fatty acids. Within a few hours, free fatty acids interfere with intracellular insulin signal transmission in skeletal muscle, reducing cells’ ability to capture glucose. This leads to the development of insulin resistance.^105^ Furthermore, the SNS (hyperactivated by CIH) stimulates the release of circulating glucagon and hepatic gluconeogenesis.^106^ CIH also induces β-cell dysfunction in diabetic mouse models, leading to insulin resistance.^107^

From a lipid metabolism perspective, CIH induces dyslipidemia by inhibiting lipoprotein lipase in adipose tissue, an enzyme responsible for lipoproteins clearance,^108^ such as very-low-density lipoprotein.^109^ CIH also increases levels of angiopoietin-like 4, an inhibitor of lipoprotein lipase.^108^ Conversely, CIH provokes increased lipid synthesis. Excess CIH-induced free fatty acids, which cannot be metabolised by tissues through mitochondrial β-oxidation due to the lack of O_2_, are reconverted into triglycerides in the liver.^108^ These accumulated triglycerides are then released into the bloodstream as very-low-density lipoprotein, leading to dyslipidemia.^108^

Regarding the interconnection between OSA and obesity, CIH induces adipose tissue inflammation through increased infiltration of proinflammatory M1 macrophages and increased expression of monocyte chemoattractant protein-1 in CIH-exposed murine models.^110^ This CIH-induced inflammation of adipose tissue triggers the NF-κB pathway and lowers adiponectin (an anti-inflammatory adipokine) in murine models, leading to an increased release of proinflammatory cytokines.^111^,^112^

One of the key mechanisms of CIH-induced metabolic dysfunction is liver dysfunction. CIH-exposed mouse models exhibited increased levels of HIF-1α in the liver.^113^ In addition, CIH reduced the levels of oxidative phosphorylation complexes, impairing mitochondrial function and increasing ROS production in liver tissue.^113^ In non-alcoholic fatty liver disease, CIH damages the gut barrier, resulting in increased release of endotoxins, which in turn reduces hepatic adiponectin production.^114^

The HIF-1α-TLR4-IL-6 pathway was found to be decisive in the worsening of CIH-induced liver fibrosis in murine experimental models.^115^ In turn, IL-6 accelerates the progression of non-alcoholic fatty liver disease by inducing ferroptosis in hepatocytes.^116^ Wu et al.^117^ demonstrated that CIH accelerated liver fibrosis in mouse models with non-alcoholic steatohepatitis through activation of liver angiogenesis via a HIF-1α-dependent mechanism.

In conclusion, these data suggest that CIH stimulates insulin resistance, dyslipidemia, obesity-related inflammation, and progressive liver disease through sympathetic overactivity, oxidative and inflammatory pathways.

### Potential benefits of intermittent hypoxia

Evidence from the literature indicates that intermittent hypoxia may also have beneficial effects on the cardiovascular system. Serebrovskaya et al.^118^ found that short-duration hypoxic exposures reduce blood pressure in subjects with hypertension. It proposed that mild, short-term intermittent hypoxia may stimulate endothelial cell relaxation and increase circulating NO levels, thereby inhibiting endothelial dysfunction and promoting vasodilation and angiogenesis.

In mice with myocardial infarction that were exposed to short cycles of intermittent hypoxia, collateral coronary circulation was more developed and myocardial vascularization was improved.^119^ Human studies of subjects with coronary artery disease also showed that short cycles of hypoxia-hyperoxia were associated with improved exercise tolerance and reduced serum troponin I and lactate concentrations.^120^,^121^

Conversely, experimental studies in mice have shown that short-term moderate intermittent hypoxia may protect against myocardial infarction. In fact, short cycles of intermittent hypoxia precondition myocardial cells to ischemia, protecting them in the case of future myocardial infarction.^122^ Mice with heart failure that were exposed to short cycles of intermittent hypoxia exhibited improved contractile function.^123^

Together, these data indicate that intermittent hypoxia may exert cardioprotective effects when mild and short-lived, highlighting a critical distinction between adaptive hypoxic conditioning and pathological CIH in OSA.

## Role of Carbon Dioxide in Obstructive Sleep Apnea

OSA leads not only to intermittent hypoxia but also to intermittent hypercapnia, defined as transient increases in arterial CO_2_. Though less discussed, intermittent hypercapnia is a clinically significant phenomenon in OSA pathophysiology. During apneas, the reduction or absence of airflow progressively increases arterial CO_2_ levels until the upper airway reopens and a compensatory ventilatory response is triggered, driven by the increased respiratory drive activity.^124^ The relationship between the CO_2_ accumulation and the hyperventilatory response post apnea is defined by the loop gain.^125^ Apneic episodes cause CO_2_ accumulation, and post-apnea hyperventilation reduces its levels. These CO_2_ cyclic fluctuations lead to ventilatory control instability, exacerbating OSA severity.^126^

Some animal studies suggested that transient CO_2_ elevations may stabilize respiratory control (e.g., by sensitizing central chemoreceptors).^127^ Conversely, insufficient post-apnea ventilatory responses in obese patients lead to a progressive increase in serum bicarbonate (HCO_3_^–^) levels with blunted drive activity, leading to nocturnal hypoventilation or diurnal hypercapnia.^128^ Repeated exposure to elevated CO_2_ may desensitize central chemoreceptors over time, leading to impaired ventilatory responsiveness. These OSA patients will evolve over time into the classical obesity hypoventilation syndrome (OHS) phenotype, characterized by daytime hypercapnia and a polygraphic pattern dominated by obstructive events.^129^ Other patients may first develop a rapid eye movement-predominant hypoventilation, which progressively extends to other sleep stages, leading to nocturnal hypoventilation and diurnal hypercapnia. A similar pattern could be observed in patients with moderate/severe chronic obstructive pulmonary disease (COPD) and a prevalent rapid eye movement hypoventilation and in approximately 10% of OHS patients with a hypoventilation-predominant polysomnographic pattern.^130^ While most OSA literature focuses on intermittent hypoxia, intermittent hypercapnia is also a key pathophysiological feature with distinct effects on cardiorespiratory and neurological systems. In uncomplicated OSA, hypercapnia may be transient, limited to apneas or hypopneas.^131^ CIH is associated with an increased tolerance to hypoxia by preconditioning mechanisms, which enhance OSA patients’ survival, even in severe chronic hypoxia. Similarly, intermittent hypercapnia may act as a protective mechanism during severe hypoxic episodes in OSA patients.^132^

Interestingly, studies in COPD patients have shown that chronic hypercapnia is not necessarily associated with a poor prognosis.^133^ Conversely, OHS patients exhibit greater comorbidity burden compared to OSA-only patients. This difference is mainly due to the elevated arterial partial pressure of CO_2_ (PaCO_2_) values, associated with a progressively depressed drive and deeper desaturations. In OHS patients, the severity of the disease is defined by the presence of both intermittent hypoxia and hypercapnia. To date, a direct causal role of intermittent or chronic hypercapnia in the morbidity and mortality of OHS patients has not been demonstrated. Nevertheless, elevated CO_2_ levels contribute to a depressed respiratory drive and a decreased work of breathing, worsening both hypoxia and hypercapnia.^134^

From a physiological perspective, hypocapnia may be more dangerous than hypercapnia. Indeed, the body always works to maintain pH homeostasis, and a lower CO_2_ is more difficult to compensate for than a chronic increase of CO_2_. In addition, recent studies have also reported an association between hypocapnia and increased in-hospital mortality.^135^,^136^

In conclusion, chronic intermittent hypercapnia emerges as a key but context-dependent feature of OSA, modulating ventilatory stability and influencing the transition toward hypoventilation syndromes.

### Hypercapnia on gas exchange

CO_2_ is a byproduct of aerobic cellular respiration, and PaCO_2_ reflects the balance between CO_2_ metabolic production and CO_2_ alveolar elimination. PaCO_2_ is regulated through a feedback loop involving central and peripheral chemoreceptors that modulate alveolar ventilation.^137^ CO_2_ has a key role in regulating and controlling breathing, especially during non-rapid eye movement sleep, where hypercapnic acidosis is a potent ventilatory stimulant.^128^,^138^ Glomus cells of the CB and chemosensitive neurons in the brain stem regions respond to changes in arterial pH and PaCO_2_. The response time of peripheral chemoreceptors to transient PaCO_2_ changes is more rapid compared to central chemoreceptors.^128^,^139^ Indeed, central chemosensitive neurons exert a sustained larger stimulation of drive ventilation in response to hypercapnia. In the lungs, coexisting hypercapnia and hypoxia may provide beneficial effects.^140^

In poorly ventilated alveoli, hypercapnia combined with hypoxia can reduce NO production, particularly in the lower lobes of severely obese patients, leading to local vasoconstriction and improved ventilation/perfusion matching.^141^ In patients with COPD and OSA (overlap syndrome), sustained hypercapnia during sleep, even in the hyperventilation phase post-apneic event, may protect against bronchoconstriction triggered by post-apnea hypocapnia.^142^ Sustained or mild hypercapnia promotes persistence of upper airway muscle activation and improves lung ventilation.^143^

In neonates, permissive hypercapnia can reduce mechanical ventilation-associated lung injury, decrease the incidence of bronchopulmonary dysplasia and protect against ventilation-induced brain injury. In addition, permissive hypercapnia induces cerebral vasodilation, increasing cerebral blood flow.^144^ Elevated levels of CO_2_ could also enhance alveolar surfactant function by increasing surface tension-lowering properties.^145^ Fluctuations in PaCO_2_ during sleep, particularly around arousals, can lead to respiratory instability. Post-apnea hyperventilation may transiently reduce PaCO_2_ below the apneic threshold, triggering central sleep apneas. The apneic threshold refers to the PaCO_2_ level immediately before the onset of a central apnea.^146^ While CO_2_ inhalation has been used to treat central apneas in neonates, central apneas can also be observed in adults with hypercapnic conditions.^132^

Monitoring PaCO_2_ levels in OSA patients is crucial in clinical practice. CPAP improves PaCO_2_ levels by maintaining upper airway patency and enhancing overall respiratory stability.^147^

Hypercapnia also influences hemoglobin-O_2_ affinity. Hypercapnic acidosis shifts the O_2_-hemoglobin dissociation curve to the right (Bohr effect), improving O_2_ delivery to peripheral tissues.^148^ The Haldane effect further facilitates CO_2_ transport, as deoxygenated hemoglobin increases its affinity for CO_2_ and hydrogen ions (H^+^), aiding in the removal of metabolic waste and the maintenance of acid-base homeostasis, particularly critical in brain tissue. Combined hypercapnia and hypoxic preconditioning exposure increases antioxidant levels such as superoxide dismutase and catalase, which reduce oxidative stress and prevent cellular apoptosis.^149^

Bach and Mitchell^150^ indicate that long-term depression was dependent on α2-adrenergic receptor activation, as demonstrated by its attenuation with specific receptor blockers. Their findings suggest that hypercapnia may modulate both excitatory and inhibitory respiratory plasticity, depending on the context and severity of exposure.

Collectively, hypercapnia emerges as a key modulator of ventilatory stability and gas exchange, with effects that may be protective or detrimental depending on the balance between respiratory drive, arousal threshold, and disease severity.

### Molecular alterations in hypercapnic conditions

Hypercapnic, particularly when combined with hypoxia, induces a series of molecular and cellular adaptations that may confer neuroprotection and cytoprotection (**Figure 2**).^151^ These effects are more pronounced under combined hypercapnia and hypoxia compared to hypoxia alone. Notably, studies suggest that intermittent hypercapnia exerts a greater involvement in the NF-κB pathway, antioxidant activity, inhibition of apoptosis, and maintenance of BBB integrity.^152^ The combined effect of hypercapnia and hypoxia significantly reduces the volume of brain damage in models of ischemic stroke.^153^

**Figure 2:**
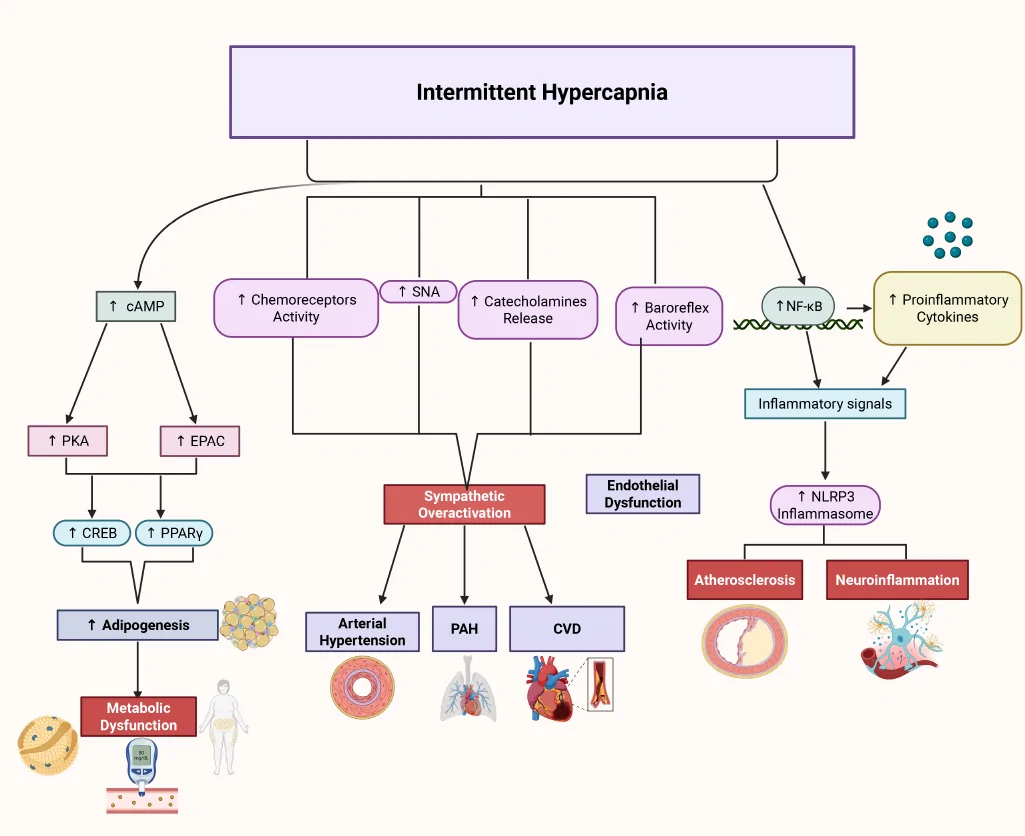
Intermittent hypercapnia from molecular pathway to clinical implication. Intermittent hypercapnia stimulates NF-κB pathway which activates NLRP3 inflammasome and proinflammatory cytokines, inducing atherosclerosis and neuroinflammation. Intermittent Hypercapnia also enhances SNA and catecholamines release, leading to sympathetic overactivation, which contributes to the pathogenesis of arterial hypertension, PAH and CVD. Finally, intermittent hypercapnia stimulates cAMP signaling pathway, activating PKA and EPAC. This cascade promotes Adipogenesis through CREB and PPARγ, ultimately contributing to metabolic dysfunction. Created with BioRender.com. cAMP: Cyclic adenosine monophosphate; CREB: cAMP response element-binding protein; CVD: cardiovascular dysfunction; EPAC: exchange protein activated by cAMP; NF-κB: nuclear factor kappa B; NLRP3: NOD-like receptor protein 3; PAH: pulmonary arterial hypertension; PKA: protein kinase A; PPARγ: peroxisome proliferative activated receptor-γ; SNA: sympathetic nerve activity.

Intermittent hypercapnia stabilizes intracellular calcium levels.^154^ Intermittent hypercapnia also promotes mitochondrial adaptations, including increased mitochondrial size and expansion of endoplasmic reticulum membranes in hippocampal neurons, which support electrolyte and calcium homeostasis. Permissive hypercapnia reduces the activity of oxidative phosphorylation and ROS production.^155^ Intermittent hypercapnia activates calcium-dependent and ATP-sensitive potassium channels.^156^ Permissive hypercapnia also increases the relative expression of mitochondrial ATP-sensitive potassium (mitoK^+^ ATP) channels, regulated by protein kinase C activation induced by increased CO_2_/HCO_3_^–^ concentrations and increased intracellular Ca^2+^.^157^,^158^ Isolated intermittent hypercapnia increases NF-κB expression, leading to elevated proinflammatory cytokine production.^159^ From a structural perspective, intermittent hypercapnia increases nucleolar number and size in nerve cells, leading to sympathetic overactivity due to enhanced synthesis of neurotransmitters, structural proteins, and neurotrophic factors (critical for neuroprotection). Permissive hypercapnia increases cellular proliferative activity with a sustained response, indicating its protective potential.^160^

Regarding apoptosis, intermittent hypercapnia exerts a significant inhibitory effect on the active form of caspase-3, reduces the cytosolic content of cytochrome C and increases the concentration of the antiapoptotic mitochondrial protein B-cell lymphoma 2 (Bcl-2).^161^ These anti-apoptotic effects have been observed both in isolated hypercapnia and in hypercapnic-hypoxic conditions, particularly in peri-infarct brain zones. Another molecular pathway modulated by hypercapnia is the maintenance of BBB integrity. Notably, hypercapnia contributes to maintaining the concentration of Evans blue dye in plasma and reducing aquaporin-4 expression.^162^ These effects lead to decreased cerebral oedema and lower BBB permeability.^163^

Another relevant molecular target of intermittent hypercapnia is HIF-1α.^164^ Several studies suggest that hypercapnia modulates the HIF-1α pathway during stress conditions. In particular, acetazolamide (a carbonic anhydrase inhibitor that elevates CO_2_ levels) has been shown to increase HIF-1α expression in the brain.^165^ While permissive hypercapnia alone may not directly change HIF-1α levels, hypercapnic hypoxia increases HIF-1α levels in hippocampal tissues, astrocytes, and the peri-infarct region.^166^ These findings suggest that intermittent hypercapnia may regulate the signaling mechanism of HIF-1 activation under stressful conditions without directly affecting its content levels. This modulation is seen as a protective mechanism against excessive HIF-1 activation, stimulating cellular adaptive potential and increasing nervous tissue tolerance.^167^

Overall, these findings indicate that intermittent hypercapnia, particularly when combined with hypoxia, activates a coordinated set of molecular and cellular adaptive mechanisms that promote neuroprotection, mitochondrial resilience, and blood–brain barrier integrity, highlighting its context-dependent role as a modulator of neural injury rather than a purely deleterious stimulus.

### Hypercapnia and the cardiovascular system

The repeated episodes of airflow obstruction during sleep produce cyclic intermittent hypoxemia and hypercapnia, which stimulate the reflex ventilatory, sympathetic, and hypertensive responses.^168^ Intermittent hypercapnia enhances sympathetic outflow, compounding the effects of hypoxia on cardiovascular risk (e.g., hypertension, arrhythmias). Indeed, intermittent hypercapnia leads to temporary sympathetic stimulation, increasing cardiac output and arousal in response to apnea.^6^ Mild hypercapnia could cause an increase in cardiac output through activation of sympathoadrenal mechanisms and sympathetic tone, increasing preload, decreasing afterload, and increasing contractility, heart rate, capacitance, and venous return.^7^ Depending on the severity, the effects of hypercapnia on the cardiovascular system could change. Hypercapnic acidosis inhibits cardiac contractility and reduces systemic vascular resistance with progression to hemodynamic instability, fatal arrhythmias, and death.^6^ On the other hand, hypercapnia can produce cardiovascular depression with direct inhibition of cardiac and smooth muscle cell contractility, independently of pH levels.^8^ Hyperoxic hypercapnia has no effect on baroreflex sensitivity but significantly increases the reflex “set point,” the baseline vascular resistance, and the mean arterial pressure. The effects of hypercapnia on vascular resistance and heart rate are sustained into the post-stimulus control period when the hypercapnic stimulus is removed. The reduced heart rate and increased mean arterial pressure during hypercapnia did not return to baseline levels in the post-stimulus control period. In fact, heart rate was further reduced and significantly lower than during the initial control period.^9^ CIH–hypercapnia in adult rats evoked hypertension and blunted the heart rate responses to acute hypoxia–hypercapnia and induced an increase in spontaneous inhibitory and decreased excitatory neurotransmission to cardiac vagal neurons.^10^ Sleep apnea may result in very high levels of sympathetic activity, which may contribute to daytime hypertension and/or precipitate cardiovascular catastrophe in hypertensive people during sleep.^11^ Interesting works in peripheral nerves of normal subjects demonstrated that hyperoxic hypercapnia causes greater increases in ventilation and sympathetic nerve activity (SNA) than hypoxia; for the same increase in ventilation, hypercapnia still causes a greater increase in SNA than hypoxia.^12^ Combined hypoxia and hypercapnia have a synergistic effect on SNA as well as on ventilation.^12^ In normal humans, both hypoxia and hypercapnia result in sympathetic nerve activation and increased sympathetic activity.^13^ Borderline hypertensive humans have exaggerated sympathetic nerve responses to hypoxia.^14^ Sleep apnea may result in very high levels of sympathetic activity, which may contribute to daytime hypertension or nondipping profile, but the role of isolated hypercapnia is not well understood.^15^ Hypoxia and hypercapnia both increase heart rate and ventilation during free-breathing conditions.^16^ Despite similar autonomic responses during free-breathing, little is known about the individual influences of hypercapnia and hypoxia compared to combined hypercapnic hypoxia on heart rate under voluntary apneic conditions. The bradycardic response to apneas after hypercapnic hypoxia is only influenced by hypoxia and not further enhanced by hypercapnia. Under apneic conditions, hypoxia rather than hypercapnia drives the cardiac component of the diving response.^169^ Acute hypercapnia can worsen pulmonary hypertension.^170^ The effects of CO_2_ on the pulmonary vasculature contrast with those in the systemic circulation. Hypercapnia can increase pulmonary vascular pressure by increasing both cardiac output and pulmonary vascular resistance. While hypercapnia increases pulmonary arterial pressure and pulmonary vascular resistance, this effect might not be as significant in preexisting pulmonary hypertension.^171^ Hypercapnia is a less potent pulmonary vasoconstrictor than hypoxia, and its more important effect may be the hypoxic vasoconstriction.^172^ While relatively little is understood about the cellular mechanism of CO_2_-induced pulmonary vasoconstriction, much of this effect appears to be catecholamine-mediated.^173^

### Hypercapnia and metabolic dysfunction

There is emerging evidence linking intermittent hypercapnia to glucose metabolism disturbances, potentially contributing to the metabolic syndrome seen in OSA patients and more common in OHS.^174^ Hypercapnia accelerates adipogenesis in both human visceral and subcutaneous preadipocytes.^175^ In contrast, hypocapnia inhibits adipogenesis.^172^ The proadipogenic effect of hypercapnia occurs independently of extracellular acidosis, O_2_ concentration or hypercapnia pattern (intermittent or sustained).^175^ Hypercapnia does not affect adipocyte hypertrophy. The mechanisms behind hypercapnia-accelerated adipogenesis involve an increased production of cyclic adenosine monophosphate (cAMP) via soluble adenylyl cyclase. This increase in cAMP leads to the activation of protein kinase A and exchange protein directly activated by cAMP (EPAC). protein kinase A and EPAC, in turn, activate proadipogenic transcription factors such as cAMP response element binding protein (CREB), CCAAT/enhancer binding protein b (C/EBPb) and PPARγ, which are key factors that trigger adipogenesis.^176^ Intermittent hypercapnia provides mechanistic clues to a pathophysiological interaction between OSA/OHS and obesity. The data suggest a vicious cycle of disease progression: OSA/OHS leads to hypoventilation, causing hypercapnia, which increases adipogenesis, leading to increased fat mass, and consequently exacerbating OSA/OHS itself.^175^ These findings support the idea that OSA itself can worsen obesity and that treatment with CPAP could help OSA or OHS patients to lose weight. CO_2_ may be a therapeutic target for OSA and OHS, and continuous CO_2_ monitoring during sleep may be important for follow-up.^176^

### Hypercapnia and neurological involvement

Permissive hypercapnia has a protective effect on the brain in hypoxic and ischemic injury.^177^ Permissive hypercapnia is a ventilation strategy in the practice of intensive care, showing protective effects in some models of neuronal damage both *in vitro* and *in vivo*.^177^ It has been demonstrated that CO_2_ at nontoxic doses exerts a protective effect on the brain during ischemia–reperfusion injury. In 2009, an experimental study showed a great increase in cerebral tolerance to ischemia with a great neuroprotective effect when hypoxia and hypercapnia are combined.^178^ In mice models, after a focal cerebral ischemia induced by middle cerebral artery occlusion/reperfusion, hypercapnic treatment significantly increased the percentage of surviving neurons and decreased the percentage of apoptotic neurons in the hippocampus.^177^ In a murine model, some authors demonstrated that the impact of permissive hypercapnia has greater neuroprotective potential compared to hypoxic conditions alone, and the same authors, in another experiment, demonstrated that hypercapnic–hypoxic training led to a 16% increase in the mean lifespan of mice.^179^,^180^ Wang et al.^181^ showed a positive correlation between awake hypercapnia and electroencephalography slowing in OHS and overlap syndrome patients, while after CPAP, awake hypercapnia decreased, and sleep electroencephalography became faster. Some studies suggest that intermittent hypercapnia may contribute to cognitive deficits and oxidative neuronal damage, although less prominently than hypoxia.^182^ Some studies found a negative correlation between electroencephalography slowing during non-rapid eye movement sleep and psychomotor vigilance in patients with OHS with severe OSA.^183^ OSA is associated with cognitive impairment and, in a few cases, with alteration in memory.^184^ Many studies, particularly in older OSA patients, have correlated hypoxia, mean SpO_2_, or arousal with cognitive impairment or with progression of neurodegenerative disorders. To date, hypercapnia predicts mild cognitive impairment only in murine models.^185^ Only patients with hypercapnia during wakefulness had worse memory performance compared with age-adjusted norms, while all groups had lower information processing speed. Additionally, higher PaCO_2_ was associated with lower Montreal Cognitive Assessment score.^162^ Electroencephalography slowing during sleep in patients with respiratory failure, OHS, and Overlap Syndrome, has been proposed to contribute to cognitive impairment.^186^ Overlap syndrome patients have been reported to show deficits in attention and executive function.^187^ Experimental studies in animals and healthy humans exposed to hypercapnia report electroencephalography slowing.^186^

In summary, although neurocognitive impairment is common in OSA, some studies demonstrated that stable daytime hypercapnia is a significant factor that prolongs reaction times in tests of cognitive function and attention and leads to specific deficits in logical memory function in obese patients with OSA, independently of subjective sleepiness, apnea–hypopnea index (AHI) or oxygen desaturation index (ODI).^188^

The autoregulatory mechanisms of cerebral blood flow are very important when cerebral damage occurs. For example, cerebral blood flow in the presence of severe hypoxia increases, which is a compensatory mechanism.^189^ The presence of hypercapnia also increases cerebral blood vessel vasodilation, not only by the molecular effects of CO_2_ on vascular smooth muscle cells, but also by NO production in endothelial cells.^190^ An increase in hypercapnia leads to an increase in sympathetic activity and blood pressure. At least during acute hypertension, hypercapnia decreases the risk of BBB disruption.^191^ In animal models, brain oxygenation increases under hypercapnic conditions, with higher O_2_ tension in brain tissue observed in both dogs and pigs.^192^,^193^ In patients with OSA, cerebrovascular reactivity to CO_2_ is increased, particularly during rapid eye movement sleep, which may play a role in cerebral blood flow regulation during periods of severe hypoxia. In these cases, hypercapnia could serve as a protective mechanism. Beaudin et al.^194^ indicated that hypercapnia occurring exclusively during sleep does not contribute to cognitive deficits in patients with severe disease; the hypercapnic load developed during sleep appears insufficient to impair cognitive function. Conversely, the same study suggests that daytime (awake) hypercapnia contributes to impaired cognitive function in individuals with severe sleep-disordered breathing. Further investigation is required to determine whether untreated hypercapnic OSA leads to progressive cognitive decline, and whether correction of daytime hypercapnia, independent of nocturnal hypoxemia and sleep fragmentation, can improve long-term neurological outcomes.

### Therapeutic effect of hypercapnia

In nature, hypercapnia is associated with longevity in some animals: whales, freshwater turtles, and naked mole-rats are all animals with underwater breathing capability, but they can tolerate long periods of apnea and hypercapnia.^195^ Naked mole-rats, known for their resistance to hypoxia and hypercapnia, also exhibit an effective antioxidant system, which some researchers consider a potential mechanism leading to the observed longevity.^196^ Many studies have shown that therapeutic hypercapnia has protective effects on lung ischemia-reperfusion injury, but its specific mechanism of action is still not fully understood. Current studies have demonstrated that therapeutic hypercapnia can alleviate lung ischemia reperfusion injury by regulating the expression of T lymphocytes.^197^ Hypercapnia, by stimulating anti-inflammatory pathways such as superoxide formation, cellular adhesion molecules, inhibiting xanthine and O_2_ radical formation, may protect cells against apoptosis during ischemia.^198^

Protective ventilatory strategies involving permissive hypercapnia are widely used in patients with severe respiratory failure, particularly in acute respiratory distress syndrome, status asthmaticus, COPD, and neonatal respiratory failure. The physiologic effects of hypercapnia are increasingly well understood, and important recent insights have emerged regarding the cellular and molecular mechanisms of action of hypercapnia and acidosis.^199^ Acute hypercapnic acidosis is protective in multiple models of non-septic lung injury.^200^ These effects are mediated in part through inhibition of the NF-κB pathway. Hypercapnia-mediated NF-κB inhibition may also explain several deleterious effects, including delayed epithelial wound healing and decreased bacterial killing, which have been demonstrated to cause worse lung injury in prolonged untreated pneumonia models.^201^ Understanding the role of hypercapnia may influence the design of OSA therapies that go beyond hypoxia mitigation.

## Molecular Mechanisms of Therapeutic Effects of Mild Hypercapnia

As discussed in previous sections, the neurological, cardiovascular, and metabolic benefits of intermittent hypercapnia have been deeply investigated, revealing potential therapeutic properties (**Table 1**).^131^,^132^,^148^,^167^,^202^,^203^,^204^,^205^,^206^,^207^,^208^,^209^,^210^,^211^ From a neurological perspective, the neuroprotective effects are mediated by three main molecular mechanisms. First, hypercapnia stabilizes intracellular calcium homeostasis, a critical process for neuronal survival, through enhanced activity of Ca^2+^-ATPase channels in the plasma membrane and mitochondria, as well as upregulation of sodium-calcium exchanger genes under ischemic conditions.^206^ Second, hypercapnia modulates the activity of Na^+^/H^+^ and Na^+^/K^+^/Cl_2_^–^ transporters, contributing to the maintenance of intracellular pH and osmolality.^209^ Third, hypercapnic conditions induce structural adaptations increasing mitochondria and endoplasmic reticulum size, which further support electrolyte homeostasis, contributing to ischemic tolerance.^167^ From a cardiovascular perspective, hypercapnia increases coronary blood flow via direct vasodilation of coronary vessels and modulation of vascular tone.^212^ In parallel, it enhances myocardial oxygen delivery by both increasing coronary perfusion and facilitating hemoglobin-oxygen unloading.^212^ Autonomic responses to elevated arterial CO_2_ levels may further support cardiac output, although the direct effects of hypercapnia on myocardial contractility are complex and context dependent.^210^ Collectively, these mechanisms suggest that controlled mild hypercapnia may confer potential functional cardiovascular benefits in selected physiological or clinical contexts, such as stress testing or critical care perfusion strategies. Regarding the molecular mechanism of metabolic adaptation, hypercapnia modulates cellular energy metabolism under hypoxic stress by enhancing glycolytic efficiency and increasing ATP retention.^213^ Additionally, it reduces ROS generation, upregulates the expression of glucose transporters, and activates hypoxia-responsive pathways, including HIF-1α and protective proteins like Bcl-2.^213^

**Table 1:** Experimental and clinical evidence of protective effects of mild controlled hypercapnia in OSA

	Benefit	Mechanism	Reference
Ventilatory control	Stabilization of breathing; reduction of obstructive and central apneas	PaCO_2_ maintains apneic threshold, reduces loop gain, and enhances respiratory motor output	132 202 203
Upper airway stability	Reduces upper airway collapsibility during sleep	Sustains activation of upper airway dilator muscles (e.g., genioglossus)	202 204
Gas exchange	Improves tissue oxygen delivery	Bohr effect (rightward shift of O_2_–Hb dissociation curve); Haldane effect	148
Cerebral circulation	Increases cerebral blood flow and oxygenation	CO_2_-induced vasodilation; NO-mediated endothelial effects	205
Neuroprotection	Reduces neuronal injury during hypoxia–ischemia	Stabilization of intracellular Ca^2+^; mitochondrial and ER adaptations; reduced ROS	167 206
Antiapoptotic effects	Increases neuronal and cellular survival	↑ Bcl2; ↓ Bax, cytochrome c and caspase3	207 208
Blood–brain barrier integrity	Preservation of BBB integrity; reduced cerebral edema	Reduces AQP4 expression; maintains endothelial function	208 209
Cardiovascular system	Improves myocardial oxygen delivery	Coronary vasodilation; mild sympathoadrenal activation	210
Oxidative stress	Reduces oxidative damage	Upregulation of antioxidant enzymes (SOD, catalase); reduces ROS production	131
Metabolic adaptation	Improves tolerance to metabolic stress	Enhances glycolysis; ATP preservation; HIF1α modulation	211

AQP4: Aquaportin-4; ATP: adenosine triphosphate; Bax: Bcl-2 associated X protein; BBB: blood–brain barrier; Bcl-2: B-cell lymphoma 2; ER: endoplasm reticulum; Hb: hemoglobin; HIF-1α: hypoxia-inducible factor-1α; NO: nitric oxide; O_2_: oxygen; OSA: obstructive sleep apnea; PaCO_2_: arterial partial pressure of carbon dioxide; ROS: reactive oxygen species; SOD: superoxide dismutase.

In conclusion, mild hypercapnia acts as a context-dependent modulator capable of promoting cellular resilience across neural, cardiovascular, and metabolic systems, leading to potential therapeutic properties.

### Therapeutic effects in ischemic and hypoxic disease

Mild hypercapnia in patients with ischemic and hypoxic diseases, including neurological and cardiologic ischemic conditions, may exert beneficial therapeutic effects. Therapeutic hypercapnia, defined as a controlled mild elevation of arterial CO_2_ levels, has been shown to reduce neuronal apoptosis after cerebral ischemia-reperfusion injury.^211^ This effect is mediated through the regulation of apoptosis-related proteins, such as increased expression of Bcl-2 (anti-apoptotic) and reduced levels of Bcl-2-like protein 4 apoptosis regulator (Bax), cytochrome c, and caspase-3, leading to improved neuronal survival.^211^ In addition, mild hypercapnia induces cerebral vasodilation, leading to reduced cerebrovascular resistance, increased cerebral blood flow, and enhanced oxygen delivery to hypoxic brain tissue.^207^ Mild to moderate hypercapnia has also been shown to preserve BBB integrity during hypoxic–ischemic conditions, reducing cerebral edema and protecting vascular function.^205^ Furthermore, respiratory training with controlled hypercapnic hypoxia improved neurological function in patients recovering from ischemic stroke, suggesting potential rehabilitation benefits.^208^

Overall, these findings indicate that controlled mild hypercapnia may represent a promising, context-dependent therapeutic strategy to enhance neuroprotection, cerebral perfusion, and functional recovery in ischemic and hypoxic conditions.

### Physiological rationale for the protective effects of hypercapnia in obstructive sleep apnea

The physiological rationale underlying the protective or deleterious effects of mild hypercapnia in OSA patients is closely related to physiological breathing control (**Table 2**).^132^,^202^,^204^ Several studies have demonstrated that enhancement of respiratory motor output through sustained hypercapnia, achieved via continuous dead space rebreathing, was effective in reducing obstructive apnea frequency in responder patients with OSA.^202^,^214^ The physiological rationale of hypercapnic rebreathing in reducing the number of apneas is linked to the physiological role of loop gain in OSA. Loop gain reflects the stability of the respiratory control system and defines the relationship between the disturbance (changes in arterial CO_2_ levels) and the ventilatory response elicited to correct that disturbance.^215^ Patients with elevated loop gain are predisposed to OSA due to an excessive ventilatory response to apneic events, leading to post-apnea hyperventilation. This overshoot in ventilation results in large intrathoracic pressure swings and increased upper airway collapsibility, leading to recurrent obstructive events.^146^ Moreover, the decrease of arterial CO_2_ levels secondary to hyperventilation reduces respiratory drive to both inspiratory muscles and upper airway dilator muscles, impairing effective airway reopening.^132^ Experimental models have shown that hypercapnic rebreathing in patients with higher loop gain attenuates the post-apneic hyperventilation phase, stabilizes breathing patterns, and reduces the frequency of apneas and hypopneas.^214^ Sustained mild hypercapnia also promotes persistent activation of the genioglossus muscle, stabilizing the upper airway during sleep.^203^

**Table 2:** Physiological mechanism of mild controlled hypercapnia in OSA

	Physiological mechanism	Evidence summary	Reference
Reduction in AHI	Increases respiratory motor output and stabilization of ventilatory control via CO_2_ chemoreceptor stimulation.	Experimental elevation of arterial CO_2_ (~3–4 mmHg) reduces obstructive events in OSA patients.	202
Enhanced ventilatory drive	Central and peripheral chemoreceptor activation increases tidal volume and breathing stability.	Hypercapnia counteracts ventilatory undershoot following arousals.	132 202
Upper airway muscle patency	LTF of pharyngeal dilator muscles, especially when combined with intermittent hypoxia.	Sustains or mild hypercapnia promotes persistence of genioglossus activation.	204
Reduced upper airway collapsibility	Increases neuromuscular activation, improves upper airway stiffness.	Observes reductions in critical closing pressure in experimental models.	202 204

AHI: Apnea–hypopnea index; CO_2_: carbon dioxide; LTF: long-term facilitation; OSA: obstructive sleep apnea.

Together, these findings suggest that mild, sustained hypercapnia may improve ventilatory stability and upper airway patency in selected OSA phenotypes by modulating loop gain and maintaining respiratory drive, highlighting its potential role as a possible therapeutic approach in OSA.

## Role of Nitric Oxide in Obstructive Sleep Apnea

OSA is associated with several systemic comorbidities,^204^ with effects that are not limited to nighttime hours but also have repercussions during the day, influencing neurovegetative regulation and vascular homeostasis.^216^

Among the mediators involved in the pathogenesis of OSA, NO plays a central role. NO is a small gaseous molecule produced endogenously through the oxidation of L-arginine by NOS. It performs numerous biological functions, including vasodilation, regulation of sympathetic tone, neuronal transmission, and inhibition of inflammatory and thrombotic activity.^216^,^217^

Therefore, understanding the role of NO in OSA, exploring aspects ranging from its production to its cardiovascular impact and responses to therapy, could be crucial to better understanding the regulatory mechanisms underlying OSA and improving the management of the disease.

### Physiology of nitric oxide in the airways and vascular system

NO is a highly reactive gaseous molecule synthesized endogenously through the conversion of the amino acid L-arginine into L-citrulline, a reaction catalyzed by three different isoforms of the NOS enzyme: the endothelial isoform (eNOS), the neuronal isoform (nNOS), and the inducible isoform.^17^,^216^ While eNOS and nNOS are constitutively expressed and primarily regulate the basal production of NO required for normal cellular function, inducible NOS is mainly activated under pathological conditions, such as inflammation and infection, producing much higher levels of NO for prolonged periods.^17^,^216^

In the respiratory system, NO is produced throughout the entire airway, from the nasal cavities to the alveoli, performing multiple crucial physiological functions. In particular, the respiratory epithelium of the upper airways (nasal cavities, paranasal sinuses, nasopharynx, and pharynx) represents one of the main sites of NO production, due to the abundant presence of nNOS and eNOS isoforms.^18^ This nasal production of NO, commonly measured via the nNO parameter, not only contributes to the regulation of upper airway patency through local vasodilatory effects but also plays a role in local immune mechanisms, such as modulation of ciliary function and defense against inhaled microbial agents.^17^,^18^

At the bronchial level, the presence of nNOS and eNOS allows for fine and continuous control of airway smooth muscle tone. Through local generation of NO, a direct bronchodilator effect is achieved, helping to maintain optimal caliber of the bronchi and bronchioles and facilitating pulmonary ventilation.^17^,^18^ At the alveolar level, NO production via the eNOS isoform is particularly relevant for the regulation of pulmonary blood flow, acting as a potent vascular modulator and consequently optimizing gas exchange, and thus appears to prevent PAH.^17^

In the cardiovascular system, the vascular endothelium is a key site for NO synthesis, primarily mediated by the eNOS isoform. eNOS-derived NO continuously regulates systemic vascular tone, maintaining vessels in an appropriately dilated state, and also exerts anti-aggregatory and anti-thrombotic functions, counteracting thrombus formation and platelet adhesion.^216^,^217^ Therefore, adequate endothelial production of NO is essential to prevent endothelial dysfunction, a condition recognized as a major risk factor for cardiovascular diseases.^217^

In light of this evidence, the evaluation of this molecule may have important implications in the management of various diseases, not only respiratory ones.

In the respiratory field, the measurement of exhaled NO is certainly a non-invasive tool for assessing the synthetic and functional activity of this mediator. In fact, the most commonly used parameter is fractional exhaled NO (FeNO), which involves single-flow determination of total exhaled NO and is primarily used as a general marker of airway inflammation.^18^,^19^

However, recent approaches have made it possible to more precisely distinguish the anatomical origin of exhaled NO using multi-flow techniques:

-Jaw NO (J’awNO): Measures the amount of NO predominantly produced at the bronchial level and provides specific insights into bronchial inflammation and dysfunction.^20^

-Concentration of alveolar NO (CANO): Reflects the alveolar concentration of NO and serves as a useful parameter to assess alveolar endothelial function and the presence of changes related to pulmonary perfusion and alveolar endothelial damage.^20^

-Nasal NO (nNO): Specific to NO production in the nasal cavities, it is mainly used as an index of local inflammation in the upper airways (particularly at the nasal and paranasal levels).^18^,^19^

These non-invasive parameters for assessing NO production and metabolism (FeNO, J’awNO, CANO, and nNO) open up interesting research and management scenarios for monitoring numerous diseases in which there is a significant alteration in NO, such as OSA.^20^,^216^,^217^

### Pathogenetic mechanism of nitric oxide in obstructive sleep apnea

Alterations in NO metabolism in patients with OSA reflect a complex and interconnected network of molecular processes, primarily triggered by the intermittent hypoxia, as summarized in **Figure 3**.

**Figure 3:**
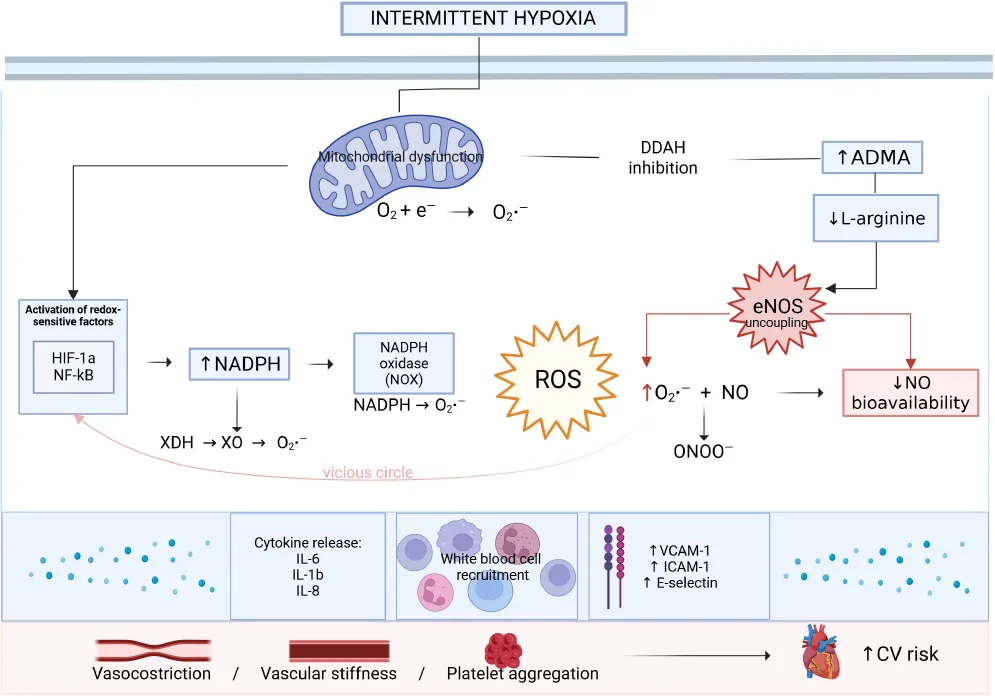
The NO molecular pathway associated with OSA increases cardiovascular risk. Intermittent hypoxia, a hallmark of obstructive sleep apnea, induces mitochondrial dysfunction and activates redox-sensitive transcription factors, including HIF-1α and NF-κB. These pathways promote the activation of NADPH oxidase and xanthine oxidase, leading to increased generation of ROS, particularly O_2_·^−^. In parallel, inhibition of DDAH results in the accumulation of ADMA and reduced availability of L-arginine, favoring eNOS uncoupling. Superoxide rapidly reacts with NO to form ONOO^−^, further reducing NO bioavailability and amplifying nitrosative stress in a self-perpetuating vicious cycle. The resulting endothelial dysfunction triggers the release of pro-inflammatory cytokines, leukocyte recruitment, upregulation of adhesion molecules, and the development of a vasoconstrictive and pro-thrombotic phenotype, ultimately increasing cardiovascular risk. Created with BioRender.com. ADMA: Asymmetric dimethylarginine; CV: cardiovascular risk; DDAH: dimethylarginine dimethylaminohydrolase; eNOS: endothelial nitric oxide synthase; HIF-1α: hypoxia-inducible factor-1α; ICAM-1: intercellular adhesion molecule 1; NF-κB: nuclear factor kappa B; NO: nitric oxide; NOX: nicotinamide adenine dinucleotide phosphate oxidase; O_2_^−^: superoxide anion; ONOO^−^: peroxynitrite; OSA: obstructive sleep apnea; ROS: reactive oxygen species; VCAM-1: vascular cell adhesion molecule-1; XDH: xanthine dehydrogenase; XO: xanthine oxidase.

During nocturnal respiratory abnormalities, repeated and rapid fluctuations in blood oxygenation occur, leading to alternating phases of hypoxia and reperfusion. These cycles generate conditions like ischemia-reperfusion injury observed in other pathological states.^218^

This intermittent hypoxia-reperfusion pattern represents a strong stimulus to produce ROS, mainly through NOX activation in leukocytes and vascular endothelial cells.^217^ The generated ROS creates a highly pro-oxidative environment that further promotes the activation of pro-inflammatory molecular pathways, particularly the one mediated by the transcription factor NF-κB.^217^ NF-κB activation stimulates the synthesis and release of numerous pro-inflammatory cytokines, such as TNF-α, IL-6, and IL-8, thereby amplifying both local and systemic inflammatory responses.^217^ This resulting pro-inflammatory and pro-oxidative environment directly impacts endothelial function, notably by significantly reducing NO synthesis and availability.

In this context, experimental studies in animal models have demonstrated clear downregulation of NOS isoforms (especially eNOS and nNOS) at both central and peripheral levels.^216^ Weiss et al.^216^ showed that in animals chronically exposed to intermittent hypoxia, there was a significant reduction in eNOS and nNOS expression in key areas involved in autonomic regulation, such as the hypothalamic paraventricular nucleus and the CB. These findings suggest that intermittent hypoxia in OSA not only impairs peripheral NO production but also disrupts its central regulation, contributing to chronic sympathetic overactivity and heightened chemosensitivity, two key features of OSA and its cardiovascular impact.

Beyond transcriptional downregulation of NOS, another key mechanism contributing to NO reduction is the so-called “eNOS uncoupling” phenomenon. ROS accumulation induced by intermittent hypoxia oxidizes the essential eNOS cofactor tetrahydrobiopterin, transforming the enzyme from a NO-producing synthase into a further source of ROS, thus fueling a harmful vicious cycle.^216^,^217^

Moreover, the increase in asymmetric dimethylarginine, an endogenous competitive inhibitor of NOS, further limits the availability of the substrate L-arginine for NO synthesis, worsening the functional deficiency of this mediator.^217^ These molecular mechanisms result in a marked reduction in NO availability, which is essential for maintaining endothelial and vascular health. Clinically, decreased NO availability manifests as impaired endothelium-dependent vasodilation, increased vascular stiffness, platelet activation, and elevated systemic blood pressure.^216^,^217^

Finally, stabilization of the hypoxia-inducible transcription factor HIF-1α due to intermittent hypoxia further exacerbates the production of inflammatory mediators and mitochondrial dysfunction, generating a molecular vicious cycle that amplifies the harmful effects of OSA on the cardiovascular system.^217^

In summary, the pathogenetic mechanisms linking OSA-related intermittent hypoxia to endothelial and vascular dysfunction are primarily driven by reduced NO availability, mediated by oxidative stress, chronic inflammatory activation, and NOS inhibition at both central and peripheral levels.^216^,^217^ This chain of pathogenic events seems to explain the strong association between OSA and increased cardiovascular risk.

### Nitric oxide as a potential biomarker of airway inflammation in obstructive sleep apnea

Over the past decades, numerous research groups have examined variations in the production and exhalation of NO in patients with OSA, yielding partly conflicting results that reflect the complexity of the underlying pathophysiology.^17^,^18^,^19^,^20^

Some studies suggest that FeNO levels are increased in OSA patients compared to healthy individuals, indicating that airway inflammation may play an important role in the disease’s pathophysiological framework.^21^,^22^,^23^ In particular, Chua et al.^24^ documented, in a case-control study, a significant increase in FeNO both before and after sleep in the OSA group, as well as a significant increase upon awakening compared to evening values, suggesting a clear relationship between OSA-related nocturnal abnormalities and increased NO production. This research group therefore suggested a probable link between local inflammatory processes triggered by intermittent obstructive events and increased levels of exhaled NO. Conversely, Agustí et al.^19^ found no significant differences in FeNO levels between patients with OSA and healthy controls, and Duarte et al.^219^ reported that single-flow FeNO measurements were not significantly correlated with OSA severity.

These apparent contradictions can be partially explained by methodological differences across studies, and in particular by the use of single-flow FeNO techniques, which do not allow accurate discrimination of the anatomical source of exhaled NO,^24^,^219^ which, as explained above, can be influenced by various anatomical and functional factors.

To overcome these limitations, multiple-flow NO measurements would appear to be more accurate and clinically informative. Dang-Thi-Mai et al.^20^ using these advanced techniques, demonstrated that the parameter J’awNO is significantly elevated in patients with OSA, showing a direct positive correlation with disease severity. Conversely, CANO does not appear to show a direct and linear relationship with AHI, but instead exhibits a stronger correlation with nocturnal oxygenation parameters, suggesting a close association between alveolar NO and OSA-related intermittent hypoxia with consequent oxidative stress and alveolar endothelial dysfunction.^20^

Another aspect that underscores the potential of NO as a biomarker in OSA is its rapid responsiveness to therapeutic interventions, particularly CPAP therapy. Studies have shown a significant reduction in elevated FeNO and J’awNO levels after several weeks or months of CPAP, indicating a marked decrease in upper and lower airway inflammation.^24^ This ability to non-invasively monitor treatment response makes exhaled NO measurements potentially useful tools for the follow-up of OSA patients.

Overall, compared to FeNO, the use of specific parameters such as J’awNO and CANO would seem to offer a promising approach for more accurately characterizing respiratory inflammation and alveolar endothelial dysfunction in patients with OSA. Although their role as primary diagnostic tools remains currently limited, they appear promising for identifying a “high-inflammation OSA phenotype” and for assessing therapeutic response.^20^,^24^ Further studies using standardized methodologies and advanced techniques will be necessary to confirm and solidify these promising findings in daily clinical practice.^19^,^20^,^24^,^219^

### Continuous positive airway pressure on nitric oxide dynamics

CPAP therapy represents the first-line treatment for patients with OSA,^220^ given its documented positive effects on respiratory parameters and sleep fragmentation.^24^,^221^,^222^ Furthermore, by restoring airway patency and preventing cyclical episodes of intermittent hypoxia, CPAP appears to be able to attenuate OSA-related pathophysiological alterations, such as chronic inflammation of the upper and lower airways, oxidative stress, and endothelial dysfunction, all of which are conditions underlying significant alterations in NO metabolism.^24^,^217^

Clinical studies have clearly demonstrated that CPAP is effective in reducing exhaled NO levels (FeNO and J’awNO) in OSA patients after a treatment period ranging from several weeks to months. Chua et al.^24^ in a prospective analysis of OSA patients undergoing CPAP therapy, reported that elevated FeNO and J’awNO levels measured before treatment significantly decreased after approximately 1–3 months of effective therapy. These findings support the hypothesis that the initial increase in exhaled NO in OSA reflects airway inflammation, which is progressively resolved by CPAP therapy.

In parallel, CPAP also exerts effects at the alveolar level. Fortuna et al.^23^ demonstrated that after 3 months of CPAP therapy, CANO values, initially reduced in patients with severe OSA compared to healthy controls, returned to normal. The pathophysiological mechanism underlying this result may be related to the effectiveness of CPAP in reducing oxidative stress. In fact, it has been shown that this therapy, by improving intermittent hypoxia, significantly reduces ROS production by inhibiting the activation of NOX, thus attenuating systemic oxidative stress.^217^ These molecular changes promote the restoration of eNOS activity, leading to increased systemic availability of NO with consequent improvement in endothelial function.^216^,^217^

Notably, Ip et al.^223^ further supported this hypothesis. Their study revealed that circulating levels of serum nitrites and nitrates, systemic markers of NO availability, are significantly suppressed in patients with OSA and that these levels rise substantially after just one night of CPAP therapy. This suggests a rapid, reversible impairment of NO synthesis or release in OSA, likely mediated by endothelial dysfunction. These findings suggest that the therapeutic effect of CPAP is not limited to improving respiratory and local inflammatory parameters but also extends to a broader systemic effect on cardiovascular function, largely mediated by restoration of physiological NO dynamics.^24^

## Therapeutic Targets and Strategies for Correcting Gas-Mediated Abnormalities in Obstructive Sleep Apnea

The gas-mediated perturbations in OSA activate converging pathways, including oxidative stress, inflammation, autonomic dysregulation, and endothelial dysfunction, which contribute to systemic morbidity. Consequently, therapeutic approaches can be conceptualized through interconnected targets focused on correcting these abnormalities (**Table 3**). CPAP remains the first-line therapy for correcting CIH secondary to OSA and for preventing consequences such as endothelial dysfunction, low-grade chronic inflammation, or sympathetic hyperactivity.^224^ However, CPAP does not work in all types of OSA patients, such as CPAP-intolerant ones or those with irreversible vascular remodeling. Supplementary nocturnal oxygen therapy helps to correct hypoxemia but does not solve the problem of upper airway collapse or arousals.^225^ Moreover, in some patient subsets, it can induce or worsen hypercapnia.^226^ For these reasons, it is not recommended as monotherapy. Finally, although evidence is limited to a few experimental studies and outcomes are only short-term, antioxidant therapy and the use of ROS scavengers or redox modulation may be beneficial against CIH.^227^,^228^,^229^

**Table 3:** Mechanisms, evidence and limitations of gas-targeted therapeutic strategies in OSA

Target gas	Pathway	Pathophysiological mechanism	Therapeutic strategy	Experimental and clinical evidence	Clinical implication and limitation
O_2_	CIH	Recurrent hypoxia-reoxygenation cycles activate oxidative stress, inflammation, sympathetic overactivity and endothelial dysfunction	CPAP	Robust human evidence abolishes CIH, improves blood pressure, endothelial function, inflammatory and oxidative markers	Gold standard therapy; adherence-dependent; does not fully reverse risk in all patients
			Supplemental oxygen (nocturnal)	Improves nocturnal oxygenation but does not correct airway collapse or arousals	May worsen hypercapnia; limited role; not recommended as monotherapy
			Antioxidant therapy (vitamin C, polyphenols, resveratrol)	Small human studies show acute improvement in endothelial function; preclinical CIH models show reduced oxidative damage	Evidence inconsistent; short-term studies; no outcome-driven RCTs; adjunctive only
	CIH-induced redox signaling	ROS activate NF-κB, MAPK and inflammatory cascades	ROS scavengers/redox modulation	Strong preclinical evidence (reduced carotid body plasticity, inflammation)	Translational gap; lack of OSA-specific clinical trials
	Hypoxia signaling (HIF-1α)	CIH stabilizes HIF-1α, promoting inflammation, angiogenesis, metabolic dysregulation	HIF-pathway modulation	Preclinical evidence only	No approved HIF-targeted therapy for OSA; systemic effects limit applicability
	CB hypersensitivity	CIH increases CB chemosensitivity → sympathetic overactivity, hypertension, ventilatory instability	CB desensitization/ablation	Experimental models: prevents CIH-induced hypertension; limited human proof-of-concept in resistant hypertension	Invasive; irreversible; not applicable to routine OSA care
			Pharmacological CB modulation (ET_A_ antagonists, antioxidants)	Animal models show reduced CB sensitivity and sympathetic activation	No clinical OSA trials; experimental
CO_2_	Ventilatory control instability (loop gain)	Post-apneic hypocapnia and overshoot ventilation perpetuate apneas	Carbonic anhydrase inhibitors (acetazolamide)	Human studies show reduced loop gain, reduced AHI, modest BP reduction	Phenotype-specific (high loop gain); side effects; adjunctive role
			Enhanced expiratory rebreathing space/controlled mild hypercapnia	Experimental and early clinical studies	Not widely available; requires further validation
	Intermittent hypercapnia	Modulates chemoreflexes, autonomic tone, cerebral and vascular reactivity	CPAP	CPAP improves PaCO_2_ and stabilizes ventilation	Primary benefit via airway stabilization
NO	Endothelial dysfunction	CIH and oxidative stress reduce NO bioavailability via NOS downregulation and eNOS uncoupling	CPAP	Human evidence: rapid increase in circulating NO metabolites; improved endothelial function	Strong evidence; systemic vascular benefit
			Antioxidant therapy (NO preservation)	Improves NO-dependent vasodilation acutely in OSA	Adjunctive; no long-term outcome data
			NOS substrate/cofactor support (L-arginine, BH_4_)	Biological plausibility; limited indirect evidence	Not validated in OSA; experimental
	Integrated gas imbalance	OSA represents a disorder of cyclic O_2_, CO_2_ and NO dysregulation	Phenotype-driven combination strategies	Emerging concept	Requires gas-based phenotyping; not yet clinical routine

AHI: Apnea–hypopnea index; BH_4_: tetrahydrobiopterin; CB: carotid body; CIH: chronic intermittent hypoxia; CO_2_: carbon dioxide; CPAP: continuous positive airway pressure; eNOS: endothelial NOS; ET_A_: endothelin A receptor; HIF: hypoxia-inducible factor; MAPK: mitogen-activated protein kinase; NF-κB: nuclear factor kappa B; NO: nitric oxide; NOS: NO synthase; O_2_: oxygen; OSA: obstructive sleep apnea; PaCO_2_: arterial partial pressure of CO_2_; RCT: randomized controlled trial; ROS: reactive oxygen species.

Modulation of HIF-1α, upregulated by CIH, has shown promising results in preclinical models, suggesting that it could be a future therapeutic approach.^230^

Furthermore, experimental carotid body desensitization prevents CIH-induced hypertension in animal models, but the procedure is invasive and not applicable to routine OSA care.^69^,^231^ Pharmacological modulation of carotid body activity via endothelin A receptor antagonists has shown promise in preclinical experimental models, reducing CB chemosensitivity and sympathetic activity.^232^ However, clinical data on OSA are currently lacking.

Regarding CO_2_, OSA patients with high loop gain (post-apnea hyperventilation and hypocapnia) may benefit from carbonic anhydrase inhibitors such as acetazolamide. In fact, acetazolamide has been shown to be effective in reducing AHI and blood pressure in the selected OSA phenotype with high loop gain.^233^

Experimental strategies aimed at stabilizing CO_2_ dynamics, such as enhanced expiratory rebreathing space or controlled mild hypercapnia, have demonstrated reductions in central and obstructive events in early clinical studies but require further validation before clinical use.^202^,^234^

Regarding NO dysfunction, CPAP stabilizes circulating NO levels, improving vascular and endothelial function.^24^ Furthermore, antioxidant therapy, by increasing NO bioavailability and reducing oxidative stress, may be useful in OSA, but long-term clinical data are currently lacking.^235^ Supplementation with NOS substrates or cofactors, such as L-arginine or tetrahydrobiopterin (BH_4_), is biologically plausible but remains unvalidated in OSA-specific clinical trials and should currently be considered experimental.^236^,^237^

All these findings indicate that therapy for gas-mediated abnormalities in OSA acts at multiple physiological levels. First, ventilatory control must be targeted, stabilizing the loop gain and preventing post-apneic hyperventilation. Second, therapeutic approaches targeting the CB chemosensitivity pathway by modulating autonomic and neural transmission are also needed, as these reduce sympathetic activity and therefore cardiovascular risk. Finally, it is essential to normalize circulating NO levels, as this prevents deleterious effects on the respiratory, neural, vascular, and endothelial systems.

In conclusion, the data from this review suggest an approach that considers the role of gases in the pathophysiology of OSA.

## Integrated Gas Signaling in Obstructive Sleep Apnea

Although oxygen, carbon dioxide, and nitric oxide are discussed separately for conceptual clarity, this narrative review indicates that their signaling pathways are tightly interconnected, leading to common molecular and potential targets in OSA (**Figure 4**). CIH acts as the primary upstream driver, stimulating redox-sensitive pathways (NOX-derived ROS, HIF-1α, and NF-κB) that reshape ventilatory control, autonomic regulation, and vascular function. Intermittent hypercapnia acts as a context-dependent modulator, influencing chemoreflex sensitivity, loop gain, and neural plasticity, while simultaneously interacting with hypoxia-driven oxidative stress and inflammatory signaling. Nitric oxide represents a downstream integrator of these processes, with its bioavailability reflecting the balance between hypoxic injury, hypercapnic modulation, and endothelial dysfunction. In conclusion, we believe it is essential to shift the understanding of OSA pathophysiology towards a mechanism in which CIH is associated with an interdependence between O_2_, CO_2_, and NO, shaping disease expression and severity rather than acting as independent mechanisms.

**Figure 4:**
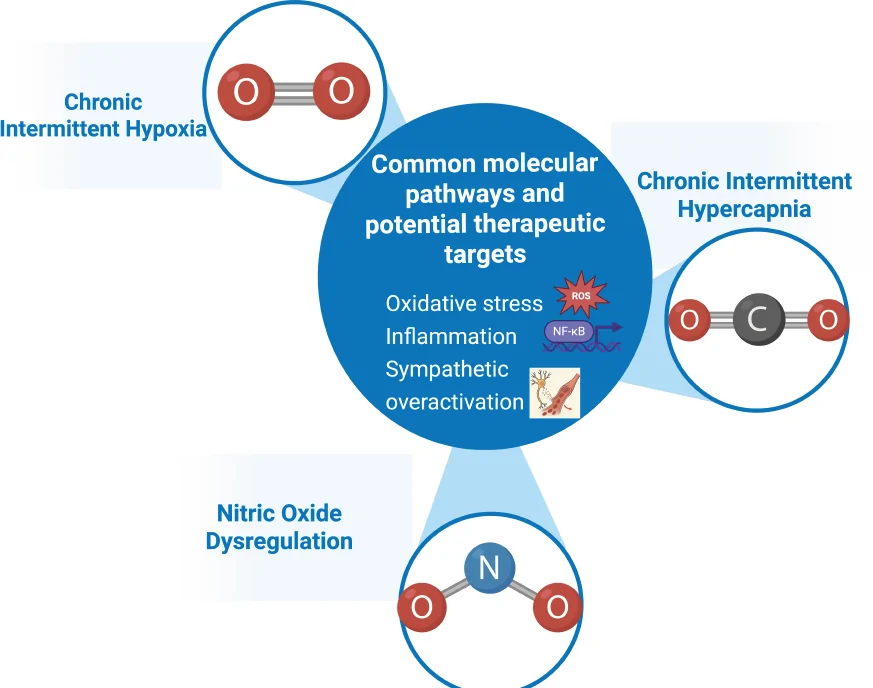
Integrated gas-mediated pathophysiology and therapeutic targets in obstructive sleep apnea. Chronic intermittent hypoxia, intermittent hypercapnia, and nitric oxide dysregulation interact and converge on common molecular pathways, oxidative stress, inflammation, and sympathetic overactivation, driving systemic consequences of obstructive sleep apnea and identifying complementary therapeutic targets beyond upper airway obstruction. Created with BioRender.com. NF-κB: Nuclear factor kappa B; ROS: reactive oxygen species.

## Limitations

This review has some limitations in terms of design and scope. First, it focuses more on the pathophysiological role of gases in OSA rather than their therapeutic role. This is mainly because current data on the therapeutic approach to gas abnormalities in OSA is only experimental and preclinical, limiting its results. Second, OSA is a highly heterogeneous disorder, and the physiological effects of gas modulation are strongly phenotype-dependent (e.g., loop gain, chemosensitivity, arousal threshold), making generalized therapeutic recommendations premature. Finally, most available studies focus on short-term physiological outcomes rather than hard clinical endpoints, underscoring the need for well-designed, phenotype-driven clinical trials integrating gas monitoring with long-term cardiovascular, metabolic, and neurocognitive outcomes.

## Conclusions and Future Directions

This literature review is the first to apply a gas-guided approach to the pathophysiology of OSA, proposing a new approach to personalizing OSA therapy. CIH provokes oxidative stress, systemic inflammation, endothelial dysfunction, and sympathetic hyperactivation, while intermittent hypercapnia contributes to ventilatory control instability, inflammation, sympathetic excitation, and potential beneficial effects, depending on the timing and intensity of exposure. With respect to other reviews, we have also shifted the focus beyond intermittent hypoxia, giving intermittent hypercapnia a leading role. Its context-dependent role makes hypercapnia unique, as it can be both a maladaptive process and a preconditioning factor for improving ventilatory stability in OSA patients. NO metabolism is impaired at both central and peripheral levels, contributing to endothelial dysfunction, cardiovascular risk, and impaired respiratory control. Future research should consider the use of tools that monitor circulating gas levels during sleep to identify specific nocturnal phenotypes.

From a therapeutic perspective, the use of mild intermittent hypercapnia or the normalization of circulating NO levels appears to be a possible future approach in the treatment of OSA. In conclusion, this review allows us to address the clinical problems of OSA and the lack of personalized treatment with gas-guided therapy.

**Facts**

1. Obstructive sleep apnea (OSA) is characterized by recurrent and interacting disturbances in oxygen, carbon dioxide, and nitric oxide signaling.
2. Chronic intermittent hypoxia, intermittent hypercapnia, and nitric oxide (NO) dysregulation activate convergent molecular, neural, and vascular pathways, including oxidative stress, inflammation, autonomic imbalance, and endothelial dysfunction.
3. Ventilatory control instability, particularly loop gain and chemoreflex sensitivity, plays a central role in shaping OSA phenotypes and clinical heterogeneity.
4. Gas-mediated mechanisms exhibit dual effects, in which maladaptive injury coexists with adaptive or preconditioning responses, especially in the context of intermittent hypercapnia.
5. Integrating gas dynamics into OSA pathophysiology provides a mechanistic framework that bridges physiology with potential phenotype-driven therapeutic strategies, while maintaining continuous positive airway pressure as the cornerstone of treatment.

**Open Questions**

1. Can gas-based physiological profiling (e.g., nocturnal carbon dioxide (CO_2_) levels, chemoreflex sensitivity, NO bioavailability) improve patient stratification and predict treatment response beyond conventional metrics, such as apnea–hypopnea index?
2. Under which conditions does intermittent hypercapnia shift from adaptive to maladaptive, and can its effects be safely and selectively modulated in humans?
3. How do neural chemoreflex pathways, particularly carotid body plasticity, interact with endothelial and inflammatory signaling in driving cardiovascular risk in OSA?
4. Can phenotype-driven, multimodal interventions targeting gas-mediated abnormalities translate into meaningful long-term improvements in cardiovascular, metabolic, and neurocognitive outcomes?

## Data Availability

*Not applicable.*
